# Correction: Lai et al. MicroRNA-21 Plays Multiple Oncometabolic Roles in the Process of NAFLD-Related Hepatocellular Carcinoma via PI3K/AKT, TGF-β, and STAT3 Signaling. *Cancers* 2021, *13*, 940

**DOI:** 10.3390/cancers14020372

**Published:** 2022-01-13

**Authors:** Chi-Yu Lai, Kun-Yun Yeh, Chiu-Ya Lin, Yang-Wen Hsieh, Hsin-Hung Lai, Jim-Ray Chen, Chia-Chun Hsu, Guor Mour Her

**Affiliations:** 1 Department of Bioscience and Biotechnology, National Taiwan Ocean University, Keelung 202, Taiwan; c.y.stephen.lai@gmail.com (C.-Y.L.); vista_jckey_1590@livemail.tw (C.-Y.L.); hearhero@hotmail.com (Y.-W.H.); 2 Institute of Biopharmaceutical Sciences, National Yang Ming Chiao Tung University, Taipei 112, Taiwan; s232579@gmail.com; 3 Institute of Biopharmaceutical Sciences, National Yang-Ming University, Taipei 112, Taiwan; 4 Division of Hemato-Oncology, Department of Internal Medicine, Chang Gung Memorial Hospital, Keelung 204, Taiwan; yehtyng@gmail.com; 5 Department of Pathology, Chang Gung Memorial Hospital, Keelung 204, Taiwan; jimrchen@cgmh.org.tw; 6 Department of Radiology, Buddhist Tzu Chi General Hospital, Taichung Branch, Taichung 427, Taiwan; jiajium@hotmail.com; 7 School of Medicine, Tzu Chi University, Hualien 970, Taiwan

The authors wish to make the following corrections to this paper [1]:

In the original article, there were mistakes in Figure 6 due to our data storage system encountering an unknown hardware error. Some image files were accidentally duplicated into other, inappropriate folders. We previously sent the wrong (old) version of the manuscript with incorrect images in Figure 6B (panel 10), 6C (panel 1) and 6D (panel 2) because the paper was undergoing a process of multiple revisions at the time of the data storage system error occurring. Now we want to correct these mistakes. However, these changes do not impact the scientific value of the article. The corrected Figure 6 appears below.

The authors apologize for any inconvenience caused and state that the scientific conclusions are unaffected. The original article has been updated.

## Figures and Tables

**Figure 6:**
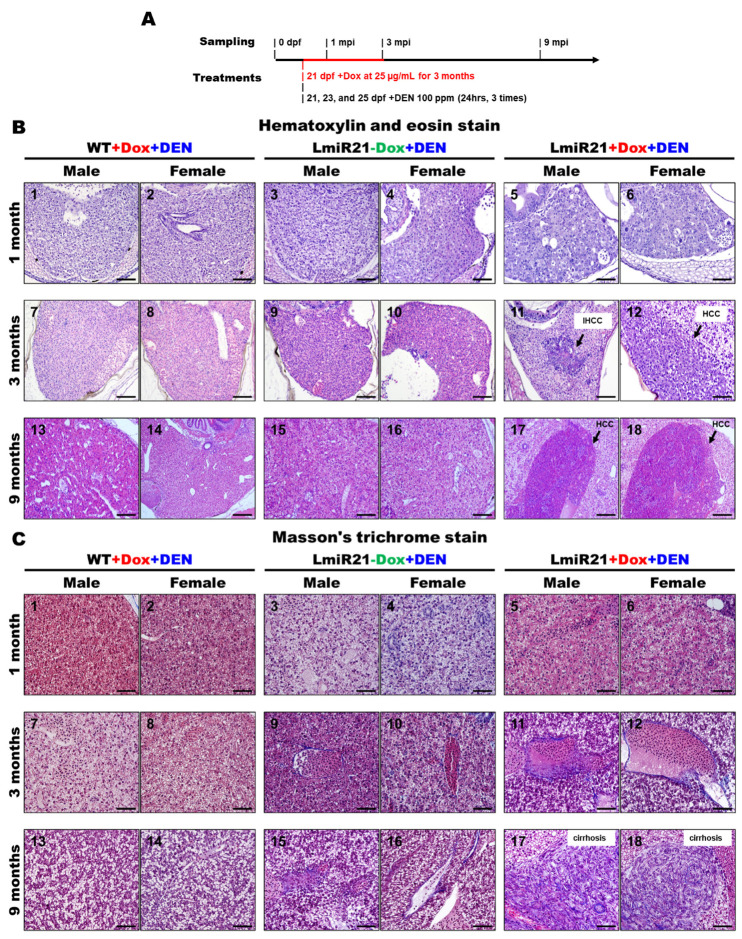

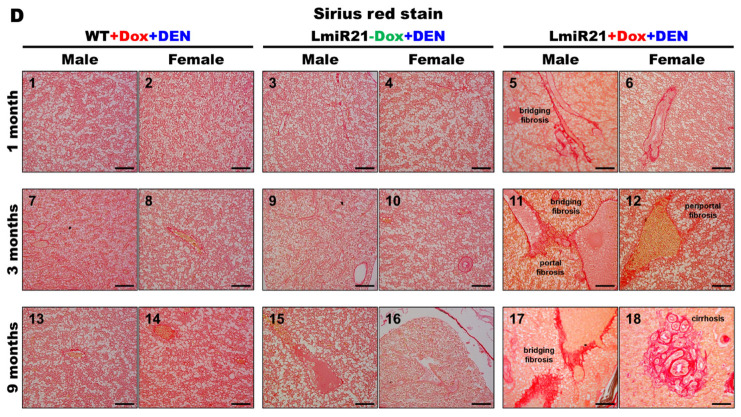
Increased susceptibility of LmiR21 zebrafish to diethylnitrosamine (DEN)-induced liver fibrosis and carcinogenesis. (**A**) Experimental design of three independent DEN treatments and doxycycline (Dox) dosing. (**B**) Representative images of livers with microscopic tumors identified following hematoxylin & eosin staining (indicated with arrows) one, three, and nine months after DEN treatment. Scale bar: 100 μm. (**C**) Representative images of livers with microscopic injuries identified by Masson’s trichome staining at one, three, and nine months after DEN treatment. Scale bar: 50 μm. (**D**) Representative images of livers with microscopic injuries identified by Sirius red staining at one, three, and nine months after DEN treatment. Scale bar: 50 μm.
